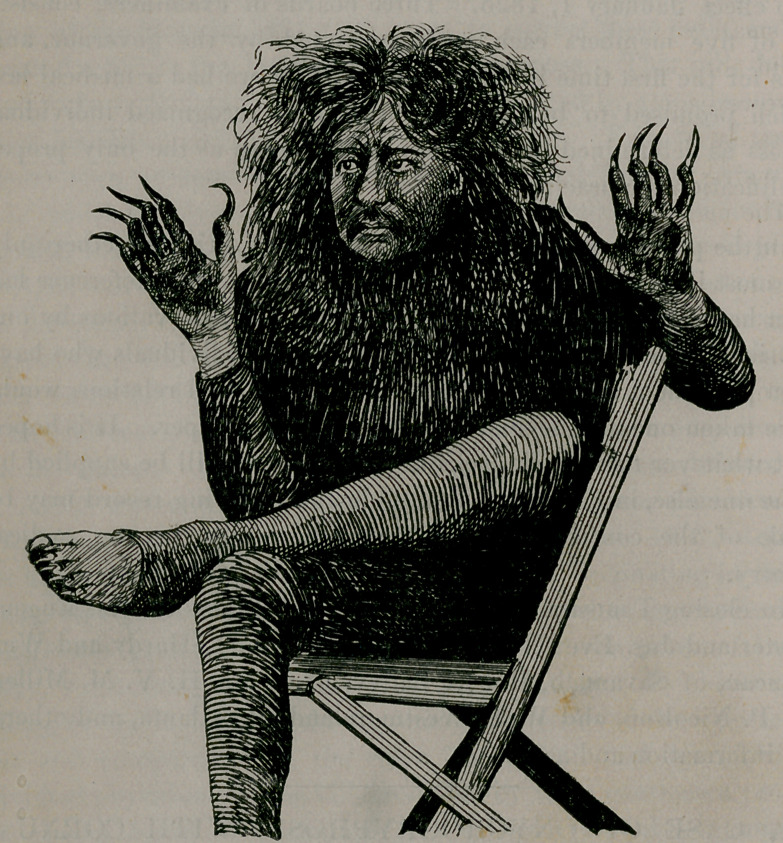# A Case of Onychogryphosis with Cornu Cutaneum

**Published:** 1895-04

**Authors:** W. H. Inghram

**Affiliations:** Atlanta, Ga.


### A CASE OF ONYCHOGRYPHOSIS WITH CORNU CUTANEUM.

BY W. H. INGHRAM, M.D.,

ATLANTA, GA.

A combination of two very peculiar as well as very rare skin diseases came under my notice in Mobile a few days ago. It was a case of onychogryphosis with cornu cutaneum, the person being a Mexican Indian posing in a show as a "wild man," the lengthened and hardened nails being advertised as claws, and the hardened places in his palms and on his feet as being caused by his walking on "all-fours" before he was captured. I was very much interested in the man and his diseases, and took a snap-shot picture of him, which is given below:

As these diseases are so rare they are little known to the average practitioner, hence it occurred to me that a brief description of them might be of interest to some readers of the JOURNAL.

Onychauxis, the term applied to any increase in the substance of the nail, may take place either as a thickening or as a general enlargement of the nail. In the second form, should it increase longitudinally it is known as onychogryphosis, and the nail sometimes grows several inches in length, curving and twisting in a very peculiar manner. In the case I speak of the nails varied in length, the longest being two and a half or three inches. They were of a dirty yellow color and were marked with longitudinal as well as transverse ribs. In every particular this was a typical case. The under surface was brownish, with irregular flaky exterior interrupted by small cavities and crossed at intervals by ridge-like projections. I did not make microscopical examination, but dare say it would have revealed in this, as in the majority of cases, a chronic state of irritation of the anterior part of the matrix and the entire area of the nail-bed.

The man may or may not have once been wild, but at any rate I could learn nothing from him as to the cause of his disease, or how long he has been affected by it.

In cases of psoriasis, chronic eczema. lichen ruber, lepra græcorum, elephantiasis arabum, etc., when extension of morbid inflammatory processes of the corium and the connective tissue of the cutis to the matrix of the nails is allowed, onychauxis may follow. In all these cases some predisposition to onychauxis must exist, as these diseases sometimes show atrophy instead of hypertrophy of the nails. The nails may be affected, too, when the skin disease is not found in contiguous parts. In spontaneous neuritis, neuralgia, chronic myelitis, traumatic lesions of mixed nerve trunks, and other neuropathic affections of a degenerative character, symptomatic hypertrophy of the nails may occur. Various chronic diseases, such as articular rheumatism, affections of the bones, or ankylosis, are sometimes followed by or accompanied with onychauxis. The disease may be congenital or acquired, the nail, in the former case, being only slightly developed beyond the normal average, at the time of birth. It grows with a greater relative rapidity, however, and the many diseases associated with papillary hypertrophy seem to favor the development of this inborn tendency.

As is readily seen, onychauxis results in the absolute loss of tactile sense to a greater or less degree, the person being unable to do any fine or delicate work, and, when the toes are affected, walking with great difficulty, and in advanced cases, being unable to walk at all. With the so-called " wild man " of whom I speak, walking was all but impossible, though the disease was not so marked on the toes as on the fingers.

It is possible to develop a healthy nail from the matrix when the hypertrophied nail is the result of a curable case of eczema, psoriasis, etc., but in the case of incurable diseases and when the nail has been affected by irremediable traumatic influences, such is impossible. When the hypertrophied product becomes a serious annoyance it may be removed by the knife, cutting pliers, or, if necessary, the saw. Whenever it is possible, the cause should be removed or remedied, whether this be eczema, psoriasis, or other disease, or some traumatism. In the former case, appropriate local remedies, ointments, rubber finger stalls, etc., should be used, and when eczema is present on the body, it will be found that arsenic, iron and other remedies suitable to this disease, will affect the nails favorably. If any form of syphilis is the cause of the diseased condition of the matrix and nail-bed, internal treatment for that disease should be used, as well as local application of powdered iodoform, mercurial ointment, or solution of corrosive sublimate, 1 to 250 of water.

An ill-fitting or tight shoe is sometimes responsible for an intensified form of onychauxis, which, if not remedied, may result in inflammation of the soft parts, and this, if neglected, may be followed by destruction of adjacent tissues, involving the tendons and bone. In cases where the toes are affected to the extent of making walking difficult, there is, from lack of exercise, a general wasting away of the body. This is true of the "wild man." He was literally "all skin and bone."

In addition to onychogryphosis he was affected with a simple form of cornu cutaneum, manifested in flattened or button-like places on the palms of his hands and on the bottoms of his feet. These places were hard, dry, and laminated, and were of a yellowish color, though in other cases they vary from gray to black, or from yellow to brown.

A more peculiar form of this disease is that seen in cutaneous horns. These are generally elongated and roundish, or conical, and may be of any size from that of a pinhead to the size and length of the finger. In structure they differ very little if at all from the normal horns found in lower animals.

They are usually single, though they may be multiple, and are often twisted and misshapen. They are commonly on the face but are seen on any part of the body, generally on elderly people but sometimes on younger persons.

If they are not injured these growths are painless. They grow slowly and drop off at times after they are of considerable size, leaving shallow ulcers, from which the horns are again developed. The American statistics showed only forty-two cases of cornu cutaneum out of 123,786 cases of skin diseases.

In treating it, the horn must be cut or twisted out, and the base cauterized with caustic potassa or chloride of zinc, the object being to prevent the reproduction of the growth.

## Figures and Tables

**Figure f1:**